# Effects of non-solvents and electrolytes on the formation and properties of cellulose I filaments

**DOI:** 10.1038/s41598-019-53215-0

**Published:** 2019-11-13

**Authors:** Ling Wang, Meri J. Lundahl, Luiz G. Greca, Anastassios C. Papageorgiou, Maryam Borghei, Orlando J. Rojas

**Affiliations:** 1 Department of Bioproducts and Biosystems, School of Chemical Engineering, Aalto University, P.O. Box 16300, 00076 Aalto, Finland; 2 Turku Centre for Biotechnology, University of Turku and Åbo Akademi University, 20520 Turku, Finland; 3 Departments of Chemical & Biological Engineering, Chemistry and, Wood Science, 2360 East Mall, The University of British Columbia, Vancouver, BC V6T 1Z3 Canada

**Keywords:** Chemical engineering, Mechanical properties, Polymers, Self-assembly, Gels and hydrogels

## Abstract

Coagulation is a critical process in the assembly of cellulose nanofibrils into filaments by wet spinning; however, so far, the role of the coagulation solvent has not been systematically elucidated in this context. This work considers organic non-solvents (ethanol, acetone) and aqueous electrolyte solutions (NaCl(aq), HCl(aq), CaCl_2_(aq)) for the coagulation of negatively charged cellulose nanofibrils via wet spinning. The associated mechanisms of coagulation with such non-solvents resulted in different spinnability, coagulation and drying time. The properties of the achieved filaments varied depending strongly on the coagulant used: filaments obtained from electrolytes (using Ca^2+^ and H^+^ as counterions) demonstrated better water/moisture stability and thermomechanical properties. In contrast, the filaments formed from organic non-solvents (with Na^+^ as counterions) showed high moisture sorption and low hornification when subjected to cycles of high and low humidity (dynamic vapor sorption experiments) and swelled extensively upon immersion in water. Our observations highlight the critical role of counter-ions and non-solvents in filament formation and performance. Some of the fundamental aspects are further revealed by using quartz crystal microgravimetry with model films of nanocelluloses subjected to the respective solvent exchange.

## Introduction

Assembling cellulose nanofibrils (CNF) into one-dimensional filaments has received increased attention, owing to the possibility to fully utilize the mechanical strength of the CNF through their alignment along the filament axis^1–11^. Wet spinning has been generally applied, transforming the aqueous CNF suspension to filaments by extrusion through a spinneret into a coagulation bath^1,4,12^. Several factors influence the filament formation and its properties, such as dope formulation^13–17^, shear rate^18,19^, and drawing ratio^13,20,21^.

The type of coagulation agent, bath concentration and temperature play important roles on filaments spun from cellulose and their characteristics, such as morphology, mechanical and viscoelastic properties^22–24^. We have previously observed that coagulation with CaCl_2_ solution facilitated wet spinning of lignin-based dopes (lignin/TEMPO-oxidized CNF (TOCNF), 70:30), while acetone was not effective^25^. Therefore, in this study, we investigated the influence of the coagulation system on the wet-spinning of TOCNF and the final properties of the spun filaments. This is a subject that has not been addressed systemically in such contexts.

Coagulation is applied in film formation (wet-casting) and fiber spinning (dry-jet or wet- spinning), where phase separation of a polymer solution occurs in the bath by mass transport and exchange between the solvent and non-solvent^26,27^. During solvent exchange, counter-diffusion occurs according to Fick’s law^24^. Thus, a polymer solution gradually loses its solvent, leading to decreased solubility^28^. In the case of the coagulation of a colloidal system such as CNF or TOCNF, instability and further phase-separation occur in the coagulation bath by the effect of interfibrillar aggregation. Two types of coagulation agents have been applied in wet-spinning of CNF, including organic solvents (such as ethanol, acetone, and tetrahydrofuran) as well as aqueous electrolytes (CaCl_2_, HCl). The organic solvents have to be miscible with water and with moderate polarity to facilitate hydrogen bonding between the fibrils^15^.

Aqueous electrolytes facilitate CNF coagulation through gelation by ion exchange^5,9,20,21,25^. The electrolyte type and concentration influence the surface charge, interaction energy and the dissociation of fibrils, consequently affecting the interfibrillar aggregation^29–32^. Moreover, the electrolytes species can displace the counterions in the charged groups (such as carboxylate), thus remarkably affecting the properties of the wet-spun filaments. So far, the effect of counterions has considered mainly aqueous dispersions for film casting. It was reported that the salt concentration, valence and size of the cations has a significant influence on the negatively charged CNF suspension (stability^33–35^, fibril orientation^36^) and the resultant film properties, such as water redispersibilty, mechanical performance, oxygen barrier performance and thermal stability^29,31,36–39^.

In this work, we systematically investigated the influence of coagulation agent (organic solvents and aqueous electrolytes) on the spinnability of TOCNF suspensions, considering the coagulation and drying times. The coagulation mechanism was studied by modelling the coagulation process through quartz crystal microgravimetry (QCM). The impact of the coagulation on ensuing filament properties was determined by characterization of the filament morphology, fibril orientation, mechanical properties, thermal stability as well as water stability and moisture sorption. Accordingly, compared to the filaments coagulated in organic solvents, those produced from aqueous electrolytes demonstrated more circular cross-section, better water/moisture stability and thermomechanical performance.

## Results and Discussion

### Impact of coagulant on wet spinning of TOCNF

**Figure 1:**
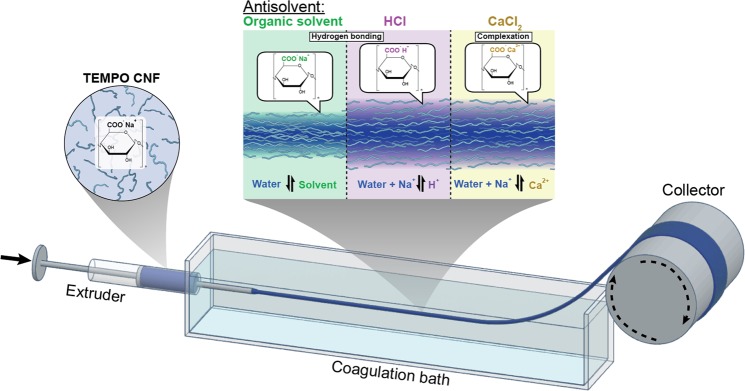
Schematic illustration from wet spinning system of aqueous suspensions of TOCNF using different spinning baths, leading to different mechanisms for filament formation.

**Figure 2:**
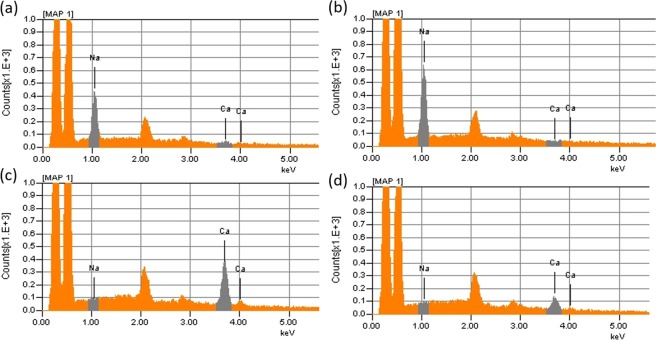
EDX spectra of the wet-spun filaments produced using different coagulants: (**a**) F_Et_, (**b**) F_Ac_, (**c**) F_Ca_ and (**d**) F_HCl_. Note: The presence of Ca in (**d**) is due to cross contamination from the tweezers used with the other systems.

TOCNF suspensions were wet spun in a spinning bath containing 400 ml of either ethanol, acetone or aqueous solutions containing NaCl, CaCl_2_ or HCl. The effects of the medium on filament formation, coagulation and drying time, washing and continuous spinnability are listed in Table 1. It took some time to coagulate TOCNF in the organic non-solvents (acetone and ethanol). After extruding TOCNF in the coagulation bath, as shown in Fig. 1, the freshly formed hydrogel thread underwent counter-diffusion between water in the TOCNF suspension and the organic coagulant (ethanol, Et or acetone, Ac). As such, TOCNF gradually lost water and coagulated. It is reasonable to assume that due to the low affinity with ethanol and acetone, a dense semi-solidified shell formed via interfibrillar aggregation, which proceeded into the core, leading to the densification of the filaments (herein, referred to as F_Et_, F_Ac_), as discussed previously^15,22^. Because of the increased density, the filaments settled in the bottom of the coagulation bath. Since water diffuses faster in acetone (differential diffusion coefficient 5.22 × 105 cm^2^ s^−1^) than in ethanol (1.222 × 105 cm^2^ s^−1^)^40^, F_Ac_ was expected to coagulate faster than F_Et_ (~5 s and 30 s, respectively). The original TOCNF was in the CNF—COO^−^Na^+^ form, which was confirmed by EDX (see sodium peak in the spectrum, Fig. S1a). In this process, the cellulose fibrils remained in the CNF—COO^−^Na^+^ form, Fig. 2a,b, indicating large amounts of sodium in both filaments, F_Et_ and F_Ac_.

**Table 1:** Effect of different coagulants on wet spinning.

Spinning bath	Coagulation time (s)	Washing	Drying time (min)	Continuous spinnability
Ethanol	≥30	No	≥12	No
Acetone	≥5	No	≥10	No
NaCl (1 M)	immediately	No	≥28	No
CaCl_2_ (1 M)	immediately	Yes	≥15 min	Yes
HCl (pH 2)	immediately	Yes	≥30 min	Yes

Note: In the case of NaCl electrolyte solution, NaCl salt precipitates on the surface of the filament. The coagulation time was estimated as the time from extrusion until filaments with a 15 cm in length were picked up from coagulation bath. ‘Immediately’ in the case of coagulation with electrolytes, is meant to indicate that such phenomenon occurred upon contact with the coagulation batch.

Unlike the case of organic coagulants, filaments could be collected immediately from aqueous NaCl, HCl and CaCl_2_ in which a dispersion-gelation transition occurred through ion diffusion^15^. In contrast, upon gelation, F_Et_ and F_Ac_ filaments tended to move to the surface of the coagulation bath. When TOCNF suspension was extruded in HCl solution at pH 2, the carboxylate groups of TOCNF became protonated (Fig. 2)^5,9^. This reduced the fibril surface charge and electrostatic repulsion, and lowered the interaction potential between cellulose nanofibrils, according to the Derjaguin-Landau-Verwey-Overbeek theory (DLVO)^29^, leading to fibril aggregation and gelation^33^. Since protons are not detectable by EDX, the reduced sodium peak in Fig. 2d indicates that the majority of carboxylate groups in F_HCl_ became protonated, i.e., in the CNF—COO^−^H^+^ form.

Several authors have shown that adding salt to CNF or CNC suspensions leads to fibril aggregation and partial gelation^29,30,32,38^. The effects of salt screening, condensation and specific interactions between counterions and COO^−^ reduce the TOCNF surface charge and further decrease the fibril interaction potential energy (DLVO), resulting in fibril aggregation. The salt concentration as well as valence play an important role in fibril aggregation. Accordingly, 1 M NaCl_(aq)_ and CaCl_2(aq)_ solutions were used as coagulants for wet spinning of TOCNF. Filaments could be immediately collected from both coagulants, though the NaCl_(aq)_ led to shorter filaments than those obtained from the CaCl_2(aq)_. This observation is explained by the weaker gel that does not support its weight when lifted from the coagulation. Upon drying, the filaments obtained from the NaCl_(aq)_ bath were covered with NaCl crystals. After placing the filaments in water, used to wash out the salt crystals, the filaments swelled significantly and became too weak to be picked up. Thus, no filaments could eventually be collected from NaCl_(aq)_.

According to Schultz-Hardy rule, the critical aggregation concentration of nanocellulose is related inversely with the valence of the cation in the electrolyte^32^. Thus, CaCl_2(aq)_ (that is, Ca^2+^) was applied as coagulant for TOCNF, inducing interfibrillar interactions by counterion condensation (Fig. 1)^21,25^. Calcium cations diffuse in the TOCNF dispersion and replace Na^+^ ^38^, leading to ionic bond formation between the negatively charged carboxylic groups and Ca^+^ (CNF—COO^−^—Ca^2+^—^−^OOC—CNF). The enrichment in calcium and depletion of sodium (EDX spectrum, Fig. 2c), observed for F_Ca_, can be taken as evidence that Ca^2+^ was exchanged with Na^+^ in the coagulation bath. We note that there was some residual chloride in F_Ca_ (Fig. S1b), indicating the possible presence of residual CaCl_2_ or other forms, such as CNF—COO^−^Ca^2+^Cl^−^ exists^31,38^. In general, to achieve filaments from TOCNF, fibril aggregation needs to occur in the coagulation bath, either by physical aggregation (organic solvent and HCl(aq)) or by chemical complexation (CaCl_2_(aq)).

The time needed for the filaments to dry mainly depended on the evaporation rate of the coagulant used for filament formation. Hence, the semi-solidified filaments obtained from ethanol and acetone (F_Et_, F_Ac_) dried much faster than those that were gellified in the electrolyte solution (F_HCl_ and F_Ca_). An approximated similar drying rate, which was assessed qualitatively, was observed for F_Et_, F_Ac_ (around 10 min). Interestingly, the gel-like F_Ca_ could not be dried in an ambient atmosphere due to the hygroscopicity of residual CaCl_2_^41^. As such, washing (by dipping the filaments in fresh water using 4 cycles and then immersion in water for 10 min and 2 h, respectively) was necessary to remove the excess salts from F_Ca_ filaments, followed by drying for 15 min. Remarkably, it took much longer time for F_HCl_ to dry (30 min), probably due to the ability of the carbonyl (COOH) groups of TOCNF to form hydrogen bonds with water^33^. Overall, gel-like filaments obtained from chemical complexation (F_Ca_ and F_HCl_) took longer time to dry than those obtained by physical aggregation/coagulation (F_Ac_ and F_Et_). In practice, this indicates a trade-off between coagulation and drying times, as the solvents that undergo fast evaporation coagulated at slower rates.

In order to increase the spinning rate, and for the purpose of filament collection, continuous spinning was performed with the help of a winder. Even though a semi-solid structure was formed in the coagulation bath, the F_Et_ and F_Ac_ filaments were difficult to handle in the continuous spinning line. In contrast, continuous spinning into gel-like F_Ca_ and F_HCl_ was successfully achieved owing to strong interfibrillar interactions. Similar results have been reported by Hagström *et al*. and Kafy *et al*. who used HCl and CaCl_2_ as coagulants for continuous spinning of TOCNF^9,21^.

### Coagulation mechanism as revealed by QCM-D

**Figure 3:**
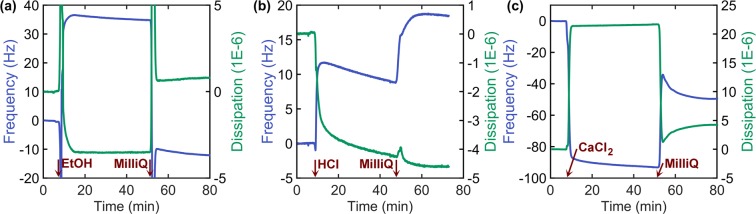
Frequency and dissipation of TOCNF-coated QCM-D crystals as a function of time upon contact with (**a**) EtOH (7^th^ overtone), (**b**) HCl_(aq)_ (3^rd^ overtone), and (**c**) CaCl_2(aq)_ (7^th^ overtone). Note: interference from bubbles is observed in the signals in (**a**), at the beginning of solvent exchange. In the time axis, the introduction of the respective solution and rinsing with MilliQ water are indicated.

In order to further understand the mechanisms involved in TOCNF coagulation, model films of TOCNF were placed in a QCM-D unit and immersed in ethanol and aqueous electrolyte solutions (CaCl_2_ and HCl). The films were fixed on a solid support pre-coated with polyethyleneimine. Thus, TOCNF fibrils were attached to the surface while, assumedly, still remaining subjected to swelling. The shift over time of the QCM frequency and dissipation are presented in Fig. 3. The Voigt viscoelastic model was used to follow the changes in mass, noting that this should be taken as an attempt to quantitatively describe the complex phenomena involved in the spinning process (see Fig. S2).

After introducing ethanol, the frequency increased (ca. 40 Hz, corresponding to QCM mass loss, Table S1) and the dissipation reduced rapidly (~4 × 10^−6^) (Fig. 3a). The results can be rationalized by the loss of associated water and its displacement with the less dense ethanol. Furthermore, as ethanol swells cellulose less effectively than water, the model film may have undergonen a reduction in thickness and associated solvent. With time, the decline in dissipation is caused by a more “rigid” TOCNF film. These observations indicate that TOCNF became “solidified” in ethanol, which can be translated, in the context of wet spinning, to the formation of a semi-solid thread/filament. In fact, after ethanol immersion, TOCNF model films absorbed more water than before (Fig. 3a). At this stage, the mass of the film increased (Table S1) compared to that at the beginning of the experiment. This result suggests that during the first immersion in water, in the beginning of the experiment, the film did not become fully swollen. Upon subsequent exposure to water, after ethanol immersion, the film absorbed more water. This effect is less relevant or absent in the case of the ethanol-coagulated filaments.

Similar effects as those discussed before took place when exposing the TOCNF film to HCl solution (Fig. 3b), though the frequency increase was limited to  ~10 Hz (indicating a mass loss, Table S1). This can be interpreted as the result of the protonation of the carboxylate groups upon exposure to the acidic environment, promoting the release of water from the TOCNF film, which became more gel-like, due to lower surface charge. Furthermore, the heavier sodium ions bound to the carboxylate groups were replaced by lighter protons, which further decreases the film weight. As the film was rinsed with MilliQ water, this trend continued, in contrast to the opposite trend observed when rinsing after ethanol immersion (Fig. 3a). This implies that the gel-strengthening effect of the acid was irreversible in neutral pH, whereas the coagulation induced by ethanol was temporal. The trends observed in the QCM-D experiments agree with the re-dispersability in water and the DVS results, discussed in other sections. Surprisingly, though, the TOCNF film underwent mass reduction (Table S1), even upon rinsing after HCl immersion (Fig. 3b). This may result from the presence of chlorine ions and associated water in the film during HCl flow.

The CaCl_2_ solution produced an effect on the TOCNF film that was in evident contrast with that observed for ethanol or HCl_(aq)_: introduction of CaCl_2(aq)_ produced a reduction of frequency, by almost 100 Hz (mass increase, Table S1). This was accompanied by an increased dissipation (by ~20 × 10^−6^, Fig. 3c). Thus, it is likely that Ca^2+^ cations diffused into the film, carrying a large number of hydration water, resulting in a net mass increase (reduced resonance frequency). The high dissipation suggests that the calcium-containing film was rather soft and hydrated (viscous). Upon rinsing with MilliQ water, the effect of CaCl_2_ was largely (but not completely) reversed (Fig. 3c), as the excess calcium ions were removed.

### Impact of coagulants on filament morphology and fibril orientation

**Figure 4:**
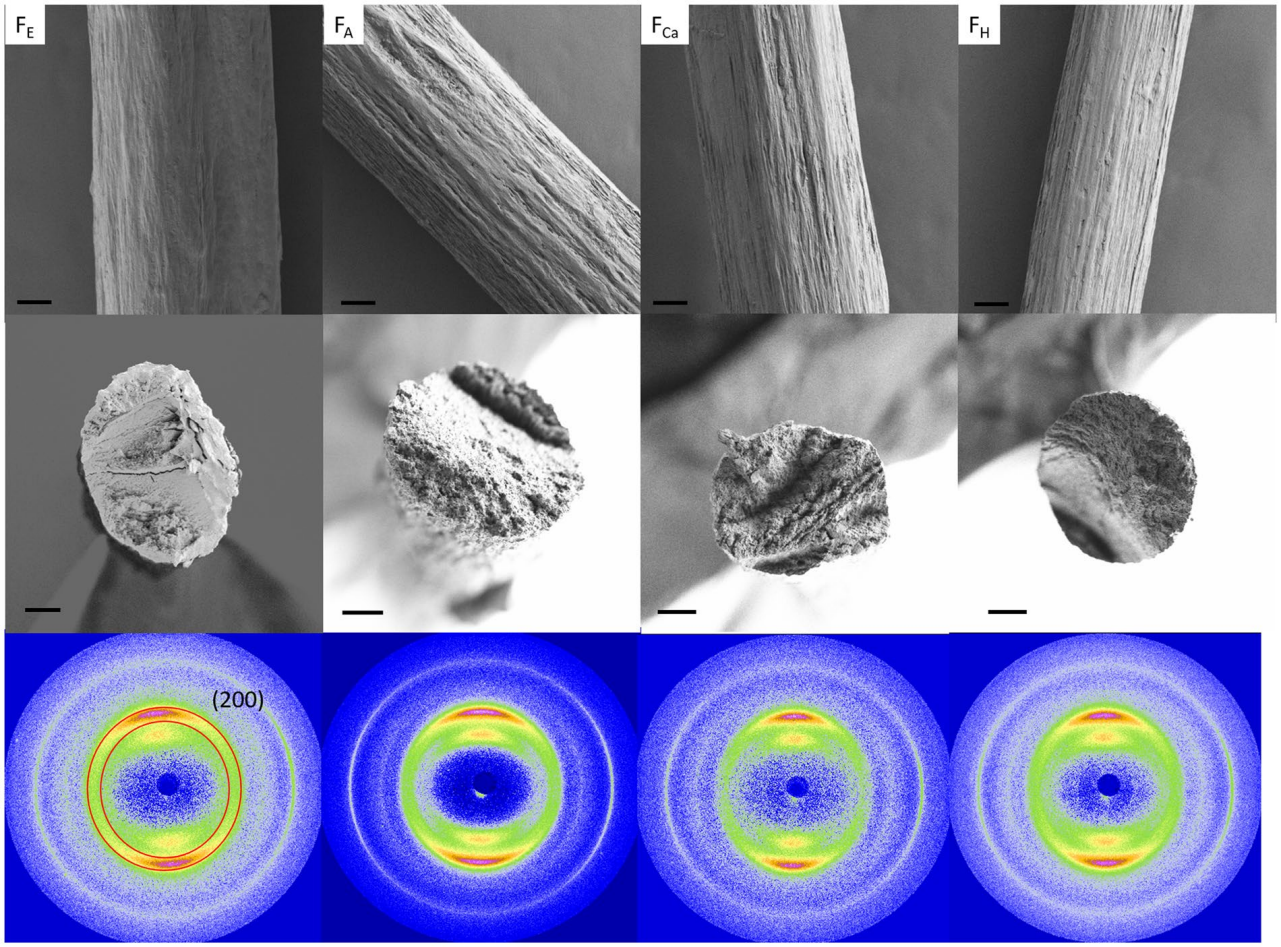
SEM images of the surface (top row, 20 µm scale bar) and cross-section at break (middle row, 20 µm scale bar) of filaments from different coagulants: ethanol (F_Et_), acetone (F_Ac_), HCl (F_HCl_) and CaCl_2_ (F_Ca_), as indicated. The bottom row includes the respective WAXS diffractograms.

SEM images in Fig. 4 (top and middle row) show that all filaments displayed a similar surface morphology as well as cross-section. However, a discrepancy was observed in the cross-section of the filaments coagulated in the organic solvents (Fig. S3). Most F_Et_ and few F_Ac_ filaments displayed irregular cross-sections. In contrast, F_Ca_ and F_HCl_ filaments were more circular. Liu *et al*. and Ziabicki *et al*. have reported that the cross-section of regenerated cellulose fibers depends almost entirely on the coagulant employed in the system^22,42^. During coagulation, a moving shell associated with the coagulant and TOCNF is formed and moves toward the center of the forming filament^15,22^. The deformation of this thin layer at the beginning of coagulation, along with the difference of mass transfer rate (water and coagulants), determine the cross-sectional shape of the resulting filament. When the mass transfer rate of water is lower than that of the coagulant (HCl and CaCl_2_ solutions), the filament swells and a circular cross section is favored. If the mass transfer rate of water is higher than that of the non-solvents (ethanol and acetone), a rigid surface layer is formed on the filament, which translates in a collapse of the cross section (filaments become thinner in the bath); hence, F_Et_ and F_Ac_ became densified and settled in the coagulation bath. The cross section of F_Et_ and F_Ac_ mainly depends on the filament deformability, before settling. In this case, F_Et_ is less dense than F_Ac_, before settling, making F_Et_ to easily deform after settling at the bottom of the bath. Thus, F_Et_ presented more irregular shapes, whereas F_Ac_ was more circular in cross section (Fig. 4). Gel-like filaments coagulated from CaCl_2_ and HCl (F_Ca_ and F_HCl_), which were displaced to the surface of the bath and kept a more circular cross section.

**Figure 5:**
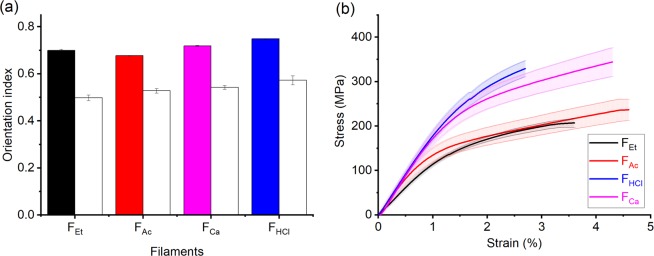
(**a**) Orientation index (filled bar) and Herman’s parameter (open bar) calculated from azimuthal data. (**b**) Tensile stress profiles for TOCNF filaments obtained by using the different coagulants. (Note: The highlighted area indicated the actual error bar in stress).

The shear forces introduced during extrusion as well as the strain applied during restraint drying favor fibril alignment^22^. Such fibril orientation (orientation index and Herman’s parameter) in the filaments was quantified by using WAXS (Table 2). The sharp arcs in the diffraction images confirm orientation along the filament axis direction (Fig. 4, bottom row). To determine the orientation of the (200) plane based on the diffractograms, the respective azimuthal profiles were plotted in Fig. S4. The peaks at 90 and 270 degrees provide further evidence that cellulose fibrils aligned along the filament axis. As shown in Fig. 5a, all type of filaments were oriented to an approximately similar extent (orientation index between 0.7–0.75 and Herman’s parameter between 0.5–0.57), which is explained by the similar shear conditions introduced during extrusion. However, some general observations seem to hold: F_Ac_ had an orientation index close to that of F_Et_ (0.7), but a slightly higher Herman’s parameter (0.53 compared 0.5). This is likely related to the faster coagulation rate, given that a shorter coagulation time allows less time for fibril relaxation in the coagulation bath^17,22^. Accordingly, F_Ca_ and F_HCl_ displayed better fibril orientation than F_Ac_ and F_Et_ (orientation indices of 0.72 and 0.75 and Herman’s parameter of 0.54 to 0.57, respectively). The results may be explained by the longer drying time and better fibril mobility during drying. Restrained drying imposes an extensional strain on the filament, as water evaporates but the filament is not allowed to shrink in the axial direction. Such extensional strain tends to orient fibrils along the filament axis. Although F_Ca_ was dried in the same way, the drying speed was much faster than that of F_HCl_ and the fibrils were already largely locked by Ca ions, which prevented the interfibrillar sliding during drying (Fig. 1).

**Table 2:** Properties of TOCNF filaments wet spun using different coagulants.

Filaments	F_Et_	F_Ac_	F_Ca_	F_HCl_
Coagulants	ethanol	acetone	CaCl_2 (aq.)_	HCl_(aq.)_
Young’s modulus (GPa)	12 ± 1^a^ (15 ± 1.4)^b^	15 ± 3^a^ (16.9 ± 2.7)^b^	17 ± 3^a^ (18.6 ± 3.7)^b^	18 ± 3.5^a^ (17 ± 2.1)^b^
Tensile strength (MPa)	207 ± 10^a^ (232 ± 13)^b^	236 ± 24^a^ (198 ± 13.8)^b^	344 ± 32^a^ (315 ± 31.7)^b^	329 ± 18^a^ (319 ± 15.2)^b^
Strain (%)	3.6 ± 0.5	4.6 ± 1.0	4.5 ± 0.7	2.7 ± 0.6
Tenacity (cN/tex)	12.6 ± 1.9	9.4 ± 0.4	16.4 ± 3.2	16.1 ± 1.2
T_onset_ (°C)	226	229	243	248
Orientation index^c^	0.7	0.68	0.72	0.75
Herman’s parameter^c^	0.5	0.53	0.54	0.57
Limiting hornification (%)	5.5	6.0	10.8	9.2

^a^Normalized against cross-sectional area obtained from micrometer, assuming a circular cross-section; ^b^Normalized against cross-sectional area obtained via SEM images; ^c^Standard deviation <10%.

### Effect of coagulant on the mechanical strength of the filaments

Obviously, the value of the cross-sectional area, determines the calculated values of tensile strength and Young’s modulus^19^. Thus, such parameter was measured using both micrometer (assuming a circular cross-section) and SEM imaging (considering the actual perimeter after image processing). Figure 5b presents the stress-strain curves normalized against the cross-sectional area obtained by a micrometer. For comparison, Fig. S5 shows the stress-strain curves normalized against the cross-sectional area from the SEM imaging. A difference can be seen in these data, as a result of the variations in cross sections, most evident in the case of F_Et_ filaments. In order to remove any influence of the determined cross-sectional area, the filament tenacity, that does not depend on the cross section, was calculated and listed in Table 2.

Regardless of the method to measure the cross-sectional area, all the data in Figs 5b, S5 and Table 2 clearly indicate that the filaments coagulated in aqueous electrolyte solutions possessed higher tensile strength and tenacity (315–344 MPa and 16.4 cN/tex for F_Ca_, and 319–329 MPa and 16.1 cN/tex for F_HCl_, respectively) than the ones coagulated with the organic solvents (207–232 MPa and 12.6 cN/tex for F_Et_, and 198–236 MPa and 9.4 cN/tex for F_Ac_ respectively). This can be explained by the fact that F_Et_ and F_HAc_, having sodium counterions, underwent reduced fibrillar bond strength and were less capable for interfibrillar hydrogen boning, thus limiting the mechanical strength^33,43^. In contrast, the carboxylate groups in F_HCl_ fibrils, which are protonated in hydrochloric acid, facilitate the hydrogen bond formation. In associated work, Benitez *et al*. showed that TOCNF films with Na as counterion had a much better modulus than those that were protonated. This is because the H^+^ introduced in the bulk dispersion results in fibril flocculation, before film formation^43^. On the other hand, the higher tensile strength of F_Ca_ can be attributed to the interfibrillar interactions caused by calcium cations, as discussed in the section ‘Impact of coagulant on wet spinning of TOCNF’. Finally, only small differences were noticed in strain at break and Young’s modulus for the different filaments, obtained either with the organic or aqueous coagulants. This can be attributed to the similar fibril orientations (Fig. 5a).

### Counterion effect on filament thermal stability

**Figure 6:**
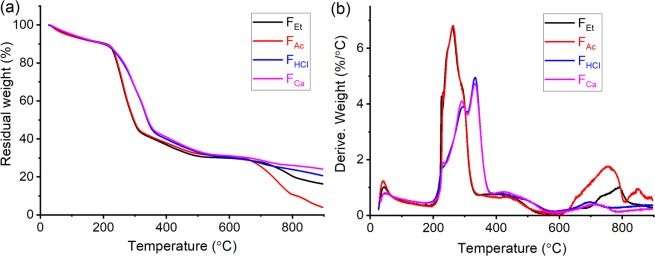
The thermal degradation curve from room temperature to 900 °C (**a**); The DTG curve of filaments from room temperature to 900 °C (**b**), for filaments obtained using different coagulants.

A similar thermo-gravimetric behavior was observed for all the filaments, within 20–900 °C (Fig. 6a). Water evaporated from the filaments at ≤100 °C, followed by cellulose chain session at 200–400 °C. Finally, the filaments were carbonized at temperatures up to 900 °C. F_Et_ and F_Ac_ (from organic coagulants) started to degrade at similar temperatures (T_onset_ of 226 and 229 °C, respectively), whereas the T_onset_ shifted to higher temperatures (248 and 243 °C) for F_Ca_ and F_HCl_, respectively (Table 2). From the derivative thermogravimetry (DTG, Fig. 6b), it can be observed that the peak corresponding to maximum degradation rate, between 200–400 °C, shifted to higher temperatures from F_Et_, F_Ac_ to F_Ca_, F_HCl_. This is because filaments obtained by coagulation from organic solvents contained a large number of sodium ions (Fig. 2a,b), which have been shown to catalyze cellulose degradation at low temperatures^44,45^. In addition, a higher residual weight was noticed for F_Ca_ and F_HCl_ compared to F_Ac_ and F_Et_. These results are consistent with Kleen’s observation that cellulose in hydrogen or calcium forms can increase the pyrolytic yield of anhydro-sugars whereas cellulose with sodium counterions would present a lower yield^46^. As such, F_Ca_ and F_HCl_ were more thermally stable than F_Et_ and F_Ac_.

### Coagulant effect on filament’s water stability/re-dispersability and moisture sorption

Different interactions with liquid were observed after soaking the filaments in water (Fig. S6). F_Et_ and F_Ac_, with Na^+^ as counter ions, swelled and turned into transparent gels immediately after immersion in water. However, no noticeable swelling was observed for F_HCl_, given that fibrils with low surface charges have limited affinity to water^18^. Similar phenomenon took place for F_Ca_, which barely swelled owing to the ionic interactions that prevented water from penetrating the interfibrillar spaces. Similar trend has been shown for TOCNF films: less water was absorbed in the presence of Ca^2+^ counterions (while intermediate and higher degrees of water swelling were recorded in systems with H^+^ and Na^+^ counterions)^31^. Dong *et al*. have reported that TOCNF films bearing Na^+^ counterions can be easily re-dispersed in water, with the help of ultrasound^36^. The re-dispersability of wet spun filaments was tested by immersion and sonication in water for 1 hour. The filaments formed with no counterion exchange (F_Et_ and F_Ac_) swelled significantly and turned into transparent gels; however, they remained cohesive and were not re-dispersed in water. In contrast, the F_HCl_ and F_Ca_ retained the filament shape, without noticeable changes after sonication.

**Figure 7:**
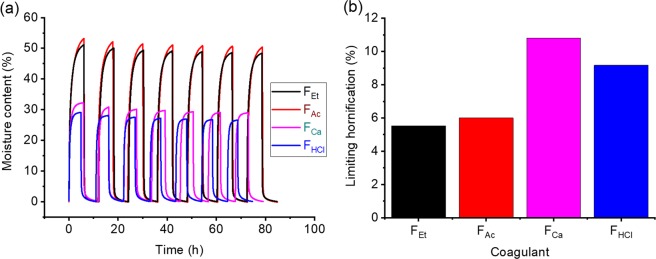
(**a**) Full moisture DVS sorption isotherms of wet-spun filaments at RH cycles between 0% to 95%. (**b**) Limiting hornification (LH) value calculated for the filaments.

The moisture sorption capacity of the filaments was followed by dynamic vapor sorption (DVS), cycling relative humidity (RH) between 0 to 95% for seven times (Fig. 7a). Clearly, in every cycle F_Ac_ and F_Et_ sorbed more moisture than F_HCl_ and F_Ca_. This finding agrees with the observation that F_H_ and F_Ca_ are more resistant to water, as discussed above. Furthermore, compared to the effect of ethanol, the results are supported by the decreased reversibility of the coagulation effect promoted by HCl and CaCl_2_, as observed in QCM experiments. As such, it can be concluded that F_H_ and F_Ca_ are the filaments that are most resistant to water, in both liquid and vapor forms, owing to reduced surface charge (F_HCl_) or electrostatic interactions (F_Ca_).

A progressive decline in the equilibrium moisture content (EMC) at 95% RH was observed after each DVS cycle. This occurs most likely due to irreversible changes occurring in the filament, e.g., “hornification”^47–49^. By fitting the EMC with an exponential function, the limiting hornification (LH) was obtained to determine the relative decrease in EMC at infinite number of cycles and compared to the first cycle (Eq. 4). The LH for each sample is presented in Fig. 7b and Table 2. Filaments coagulated in organic coagulants (F_Et_, F_Ac_) were the least hornified (LH 5–6%), most probably because of their high surface charge that inhibits the effects associated with hornification, such as irreversible hydrogen bond formation, pore closure and fibril aggregation^47^. For the other filaments (F_Ca_ and F_HCl_), the TOCNF surface charge is reduced due to electrostatic screening (CaCl_2_) or protonation (HCl), which makes these filaments more susceptible to hornification. Similar observations have been reported for the hornification of filaments wet-spun from CNF bearing different surface charge density^19^. The moisture sorption phenomenon was further characterized using the parallel exponential kinetics model (see supporting information, Fig. S7).

## Conclusions

Wet spinning of TOCNF was carried out using spinning baths comprising either organic nonsolvents (ethanol or acetone) or electrolyte solutions (NaCl_aq_, CaCl_2aq_ or HCl_aq_). Enhanced interfibrillar interactions were observed after solvent exchange (ethanol and acetone anti-solvents) or via electrostatic screening and complexation (aqueous electrolytes). A correlation between the coagulant type, filament morphology, as well as fibril orientation and the corresponding physical properties is found. The counterions of carboxylate groups present in TOCNF played a critical role in the resultant properties of the filament. The physical aggregation that occurred in the organic solvent retained fibrils in the sodium form (CNF—COONa). This yielded filaments (F_Et_ and F_Ac_) with relatively weaker mechanical strength, lower thermal and water stability, and higher moisture sorption capacity. Those formed via hydrogen bonding or chemical complexation (F_HCl_ and F_Ca_ respectively) presented the opposite characteristics. A more circular cross-section was observed for such filaments.

## Materials and Methods

### TEMPO oxidized cellulose nanofibrils (TOCNF)

Never dried bleached birch fibrils (UPM, Pietarsaari Mill) were oxidized by 2,2,6,6-tetramethylpiperidine-1-oxyl (TEMPO, Sigma-Aldrich) at pH 10. After washing with deionized water, the oxidized fibrils were passed through high-pressure microfluidization (Microfluidics Corp., USA), yielding TEMPO oxidized cellulose nanofibrils (TOCNF) of 1.6 wt% solids content and 0.6 mmol g^−1^ carboxylic groups. The TOCNF used in this work was in the sodium form.

### Wet spinning of TOCNF

Four different coagulants (ethanol, acetone, and aqueous electrolytes, HCl and CaCl_2_) were used to investigate their influence on wet spinning. TOCNF was loaded into a 50 ml syringe and extruded (10 ml/min) through a tube (44.5 cm length, 6 mm inner diameter), followed by a needle (3.7 cm length and 1.2 mm inner diameter) and finally into a the spinning bath that contained either ethanol or acetone as non-solvent or aqueous solutions containing CaCl_2_ (10 wt%) or HCl (pH 2). The drying rate was estimated qualitatively as the time lapsed soon after picking the filament from the bath until it remained straight when suspended in air and with no observable shrinkage upon removal from the holder. At least ten specimen were considered. Here, the term coagulation is used loosely since the involved phenomena can be quite complex and different than the typical solvent exchange. The collected filaments were dried in ambient conditions under restrained drying (by fixing both ends of the filaments). The drying time was recorded from the time the given filament was withdrawn from the spinning bath until it became dried, forming straight filament. The filaments collected from CaCl_2_ and HCl solutions required an extra washing steps, which were carried out by dipping the filaments in fresh water (4 times), followed by immersion in water for 10 min and 2 h, respectively. After each washing step, the filaments were dried in air, also under restricted shrinkage. The drying time was recorded from the time the filament was picked up from the bath, until the filament stayed straight in the air, without shrinking after removal of one of the two holding ends. The dry filaments obtained from coagulation in ethanol, acetone, CaCl_2_ and HCl are thereafter referred to as F_Et_, F_Ac_, F_Ca_ and F_HCl_, respectively.

### Continuous spinning

A continuous spinning test was conducted using the same setup as above with an addition of a winder (diameter of 22 cm) for filament collection. TOCNF was extruded at 0.64 m min^−1^ and the winding speed was controlled at 3.6 m min^−1^.

### Tensile strength

The effect of coagulant on the mechanical strength of the filament was determined with an Instron 5944 Single Column, Tabletop Universal Testing System, operated in tensile mode with a load cell of 5 N. At least seven specimens per sample were measured with gauge length of 2 cm and stretching rate of 2 mm min^−1^. Prior to testing, filaments were conditioned overnight at 50% humidity and 23 °C. The filament cross-sectional areas were obtained by using a micrometer, and also from imaging with a scanning electron microscope (using image-processing software ImageJ to determine the perimeter and the actual cross-sectional area). Ten specimen were measured for each type of filaments. Note: for the cross-section area obtained from SEM, an average value was obtained from at least eight specimens tested for each sample.

### Thermogravimetric analysis (TGA)

The filament thermal stability was determined under N_2_ flow using TA Instrument (Thermo Gravimetric Analyzer Q500). Filaments were cut into ~2 mm length and heated up to 900 °C from room temperature with a heating rate of 10 °C min^−1^.

### Scanning electron microscopy (SEM) and energy dispersive X-ray spectroscopy (EDX)

Filament morphology and cross sections at break were observed using Zeiss SIGMA VP (Carl Zeiss Microscopy Ltd, Cambridge, UK) at 1.6 kV with a working distance of 1 cm. Before imaging, the filaments were deposited with 5 nm-thick Pt/Pd coating. The fracture was made by bending the frozen filaments in liquid N_2_. For detection of elements (Na and Ca), EDX spectra were recorded in a JEOL JSM-7500FA SEM (Japan) at 15 kV acceleration voltage.

### Wide angle X-ray scattering (WAXS)

The fibril orientation of the filaments was determined with a MicroMax-007 X-ray generator (Rigaku, Japan). WAXS was performed in transmission mode with an X-ray wavelength of 1.54 Å, a beam size of 120 μm and an exposure time of 10 minutes. A Mar345 plate detector was employed to collect sample diffraction patterns with 200 mm distance of sample-to-detector. The filaments were aligned horizontally and placed perpendicular to the beam. Before evaluation, the background was subtracted from the diffraction patterns. Based on azimuthal intensity distribution profiles, orientation index (π) and Herman’s orientation parameter (S) were calculated according to Eqs (1) and (2):

1 $$\pi =\frac{{180}^{\circ }-FWHM}{{180}^{\circ }}$$

where FWHM is the full width at the half maximum (in degrees) of one of the two peaks in the azimuthal intensity distribution profile. π was calculated for both peaks and their average reported.

2 $$S=\frac{3}{2}\langle co{s}^{2}\gamma \rangle -\frac{1}{2}$$

Assuming cylindrical symmetry in the filament, the average cosine 〈cos^2^γ〉 was obtained from the azimuthal angle φ according to Eq. (3)^50^.

3 $$\langle co{s}^{2}\gamma =1-2co{s}^{2}\phi \rangle $$

where

$$\langle co{s}^{2}\phi \rangle =\frac{{\sum }_{{\phi }_{0}}^{{\phi }_{0}+\pi /2}I(\phi )\sin \,\phi co{s}^{2}\phi }{{\sum }_{{\phi }_{0}}^{{\phi }_{0}+\pi /2}I(\phi )\sin \,\phi }$$

here, *I(φ)* is the intensity detected at azimuthal angle *φ*, and *φ*_0_ is the azimuthal angle in the beginning of the range used for the calculation of the average cosine 〈*cos*^2^*φ*〉*. S* was calculated at *φ*_0_ of 0, π/2, π and 3π/2 and the average of these values is reported. A value of 1 for the orientation parameter indicates a fully oriented structure while 0 means a disordered structure.

### Quartz crystal microbalance with dissipation (QCM-D)

The effect of different coagulants on TOCNF was studied with the QCM-D technique using a Q-Sense E4 apparatus (Q-Sense, Sweden). Firstly, UV/ozonized QCM-D gold crystals were coated with an anchor layer of polyethylenimine (PEI) by immersing them in 0.1 g/L aqueous PEI solution for 15 min. After rinsing with MilliQ water and drying with N_2_ gas, the PEI-covered crystals were first spin-coated with MilliQ water to wet the surface (3000 rpm, 30 s) and then with a 1 g/L TOCNF dispersion in MilliQ water (3000 rpm, 60 s). Finally, the obtained TOCNF films were dried with N_2_ flow and heat-treated at 80 °C for 10 min.

After placing the TOCNF-covered crystals in the QCM-D chambers, they were rinsed with degassed MilliQ water until the frequency and dissipation signals stabilized. After ~5 min, degassed ethanol or aqueous solutions (1 M CaCl_2_ or HCl at pH 2) was introduced for ~30 min. Subsequently, the films were rinsed with degassed MilliQ water. All QCM-D experiments were conducted at least twice at 23 °C and under a constant flow of 100 μL/min.

### Dynamic vapor sorption (DVS)

Dynamic vapor sorption (DVS, Surface Measurement Systems, UK) was used to determine the water vapor sorption isotherms of the filaments. Filaments (~10 mg, ~5 mm in length) were loaded into the sample pan hanging from a microbalance in a climate-controlled chamber. Firstly, the sample was allowed to stabilize (change in mass below 0.002%/min over a period of 10 minutes) at a relative humidity (RH) of 0%. Secondly, the RH was increased to 95% and maintained steady until the sample mass stabilized, followed by decreasing the RH back to 0%. In this way, RH was cycled between 0% (moisture desorption) and 95% (moisture sorption) for seven times in total. The relative increase in sample mass compared to the mass after the first drying at 0% RH corresponds to the moisture content of the sample at 95% RH.

The changes in the equilibrium moisture content (EMC) obtained at the end of each cycle at 95% RH, reflect the effect of the so-called “hornification” on the sorption capacity of the sample^48^. The EMC as a function of the humidity cycle was fitted to an exponential function. Using this fitting, the limiting hornification (LH) was estimated according to

4 $$LH=\frac{EM{C}_{1}-EM{C}_{n}}{EM{C}_{1}}\times 100 \% ,$$

where *EMC*_1_ is the EMC at the end of the first cycle at RH 95% and *EMC*_*n*_ at the end of the n^th^ cycle when *n* approaches infinity. *EMC*_*n*_ was estimated according to the exponential fit of the EMC measured at the end of each of the seven cycles. Notably, the EMC values obtained by DVS contain the contribution of external surfaces, which are not susceptible to hornification. As such, Eq. (4) yields the limiting hornification as a percentage of total initial sorption capacity.

## Supplementary information

Supporting information
