# Supplementary material for: Effects of non-solvents and electrolytes on the formation and properties of cellulose I filaments
Source: Sci Rep. 2019 Nov 13;9:16691. doi: 10.1038/s41598-019-53215-0 (PMC6854096; doi:10.1038/s41598-019-53215-0)
Supplement: Supplementary file 1 — Supporting information [file 41598_2019_53215_MOESM1_ESM.pdf]

# Supporting Information

# Effects of non-solvents and electrolytes on the formation and properties of cellulose I filaments

Ling Wang,[1] Meri Lundahl,[1] Luiz G. Greca,[1] Anastassios C. Papageorgiou,[2] Maryam Borghei,[1] Orlando J. Rojas[*,1,3]

[1]Department of Bioproducts and Biosystems, School of Chemical Engineering, Aalto University, P.O. Box 16300, 00076 Aalto, Finland. [2]Turku Centre for Biotechnology, University of Turku and Åbo Akademi University, 20520 Turku, Finland. [3]Departments of Chemical & Biological Engineering, Chemistry and, Wood Science, 2360 East Mall, The University of British Columbia, Vancouver, BC V6T 1Z3, Canada. Correspondence and requests for materials should be addressed to O.J.R (email: orlando.rojas@aalto.fi).

This Supporting Information document contains four figures and 1 table in seven (7) pages:

Figure S1. EDX spectra of freeze dried TOCNF and $F_{Ca}$.

Figure S2. Frequency and dissipation of TOCNF-coated QCM-D crystals as a function of time with Voigt viscoelastic model.

Table S1**.** The mass changes in TOCNF coated QCM-D crystals during fluid change, based on Sauerbrey equation

Figure S3. The SEM image of $F_{Et}$ and $F_{Ac}$ showing an irregular cross-section (All the scale bar is 20 μm).

Figure S4. Azimuthal profiles of (200) reflection obtained from WAXS diagram of TOCNF filaments.

Figure S5. The filament stress-strain curves normalized against cross-sectional area obtained via SEM images.

Figure S6. Wet-spun TOCNF filaments after soaking in water for one week.

Figure S7. The time scales and equilibrium moisture contents associated with the fast and slow sorption regimes for filaments coagulated in acetone, ethanol, $CaCl_2$ and HCl.

(a)

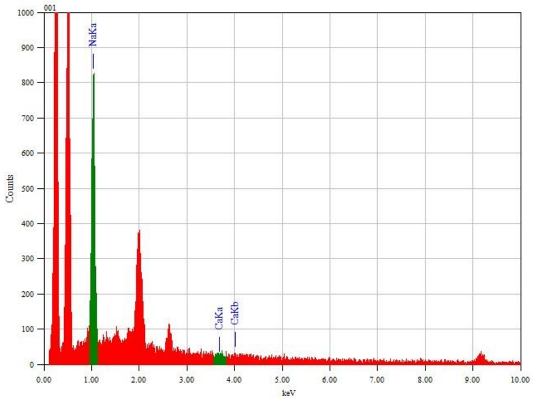


(b)

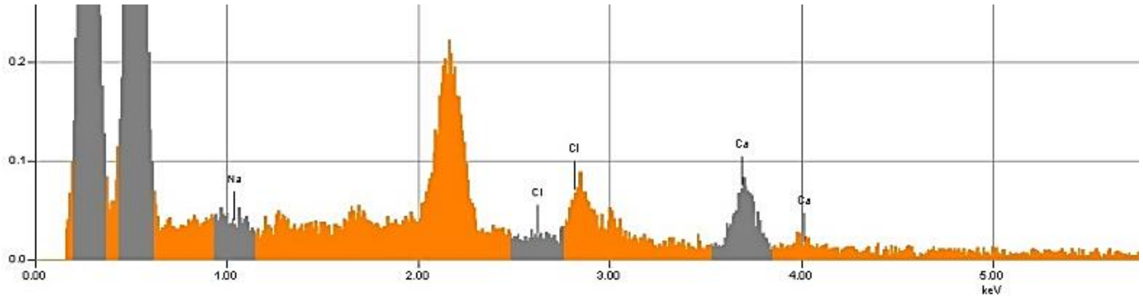


**Figure S1.** EDX spectra for (a) freeze-dried TOCNF, and (b) $F_{Ca}$ showing chloride element on the filament.

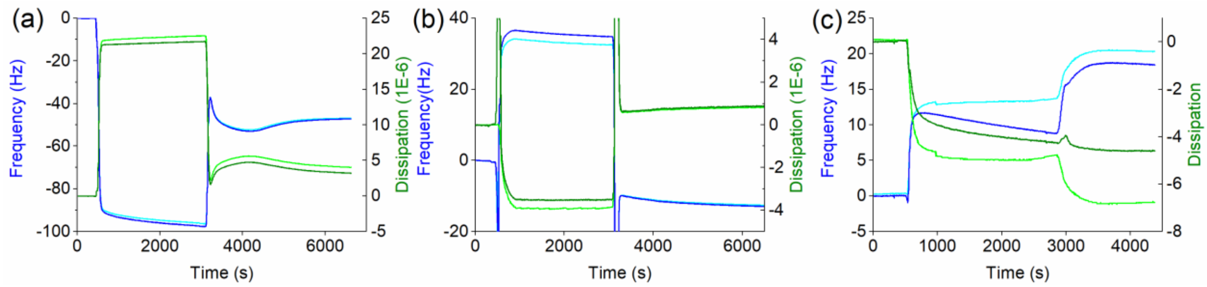


**Figure S2**. Voigt viscoelastic model on the frequency and dissipation of TOCNF-coated QCM-D crystals as a function of time upon contact with (a) EtOH (7$^{th}$ overtone), (b) $HCl_{(aq)}$ (3$^{rd}$ overtone), and (c) $CaCl_{2(aq)}$ (7$^{th}$ overtone). Note: The lighter color curve in each graph gives the Voigt fitting in the specific overtone.

**Table S1.** The mass changes in TOCNF coated QCM-D crystals during fluid change, based on Sauerbrey equation.*

| Solvent | ethanol | HCl | CaCl2 |
|---|---|---|---|
| Δm1 (solvent to replace water)/ng cm$^{-1}$ | 619.5 | 159.3 | -1628.4 |
| Δm2 (water to replace solvent)/ng cm$^{-1}$ | -849.6 | 159.3 | 761 |

*Sauerbrey equation: $\Delta m = \frac{-c\Delta f}{n}$, where $C$ is a device specific constant (17.7 ng cm$^{-2}$ Hz$^{-1}$), $\Delta f$ the change in oscillation frequency of the crystal and $n$ is the overtone used.[1]

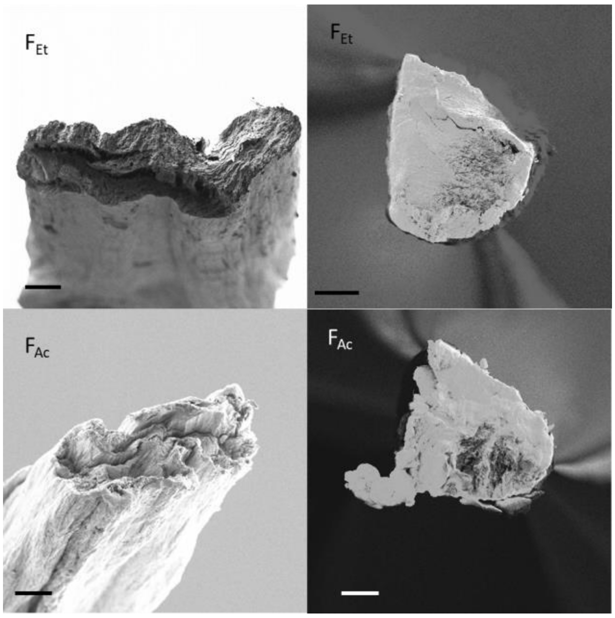


**Figure S3.** The SEM image of $F_{Et}$ and $F_{Ac}$ showing an irregular cross-section (All the scale bar is 20 μm).

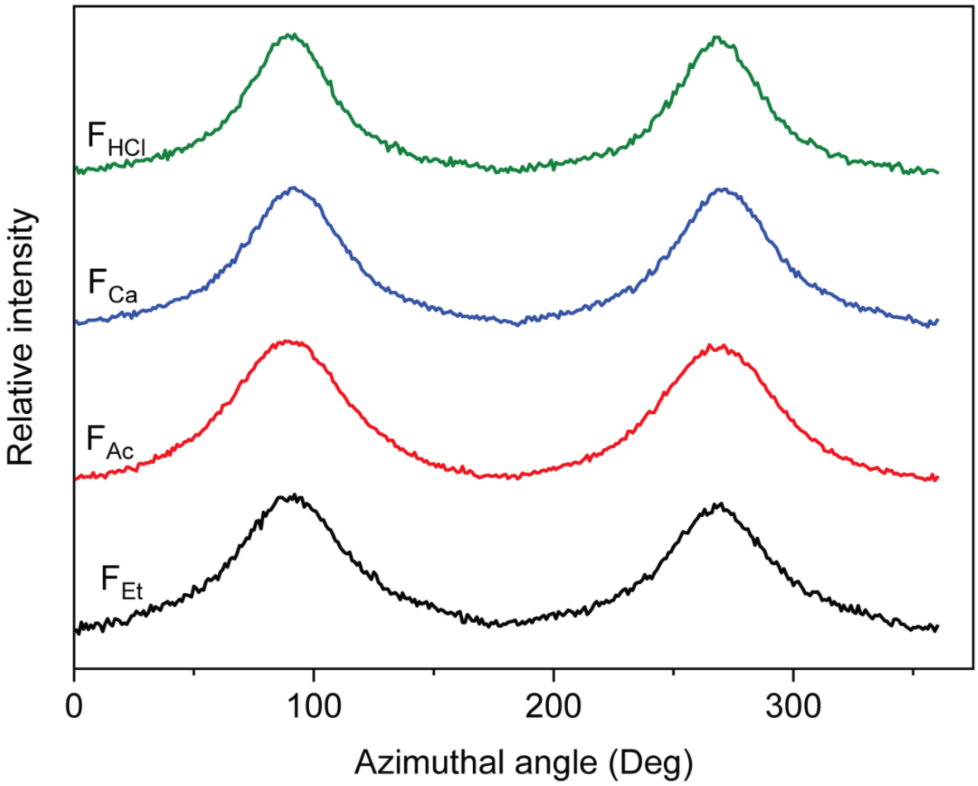


**Figure S4.** Azimuthal profiles of (200) reflection obtained from WAXS diagram of the respective filaments, as indicated.

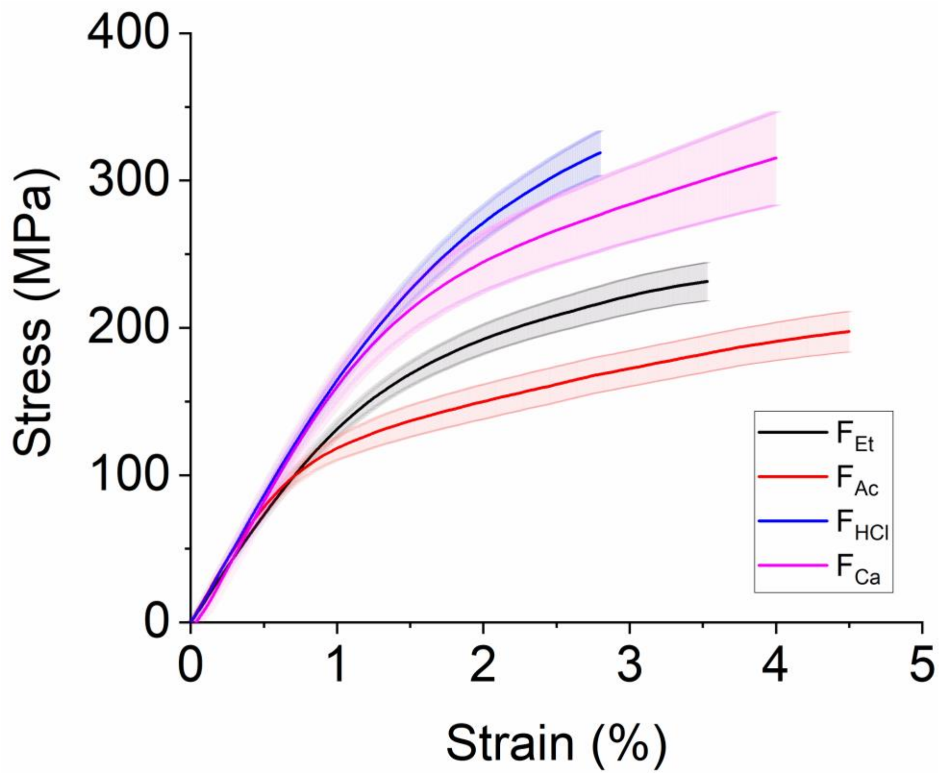


**Figure S5.** The filament stress-strain curves normalized against cross-sectional area obtained via SEM images.

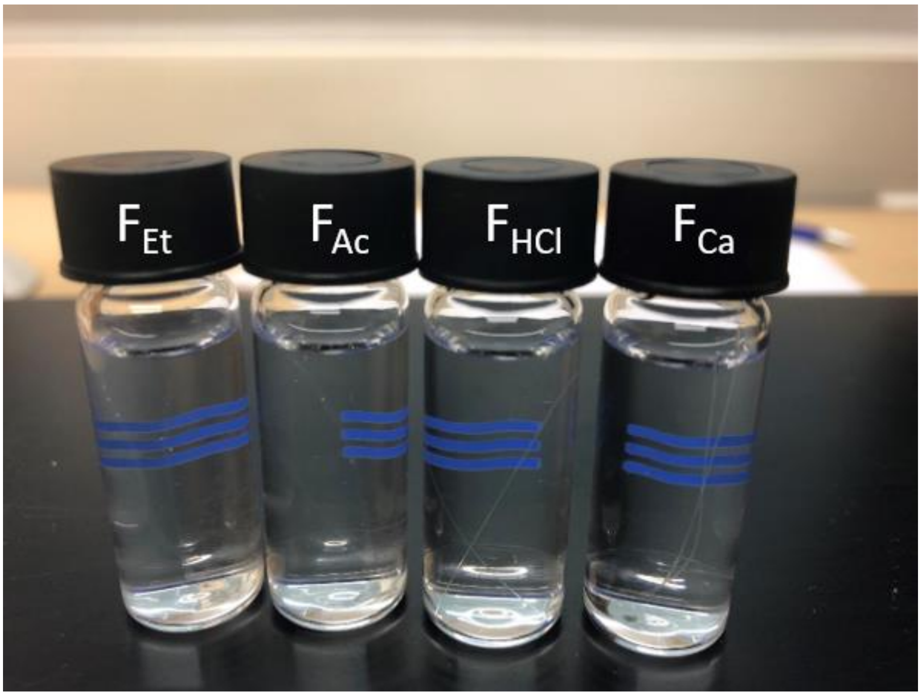


**Figure S6.** Wet-spun TOCNF filaments immersed in water for one week.

**Parallel exponential kinetics analysis of dynamic vapor sorption**

The moisture sorption isotherms measured with dynamic vapor sorption (DVS) are presented in **Figure 6a** of the main article. Based on these isotherms, the sorption phenomena were characterized by approximating each adsorption cycle with the parallel exponential kinetics model.[2] According to this model, the total moisture content (MC) sorbed by a sample consists of moisture sorbed at exposed sorption sites, where water molecules can reach fast, and at more hidden sorption sites, where water molecules reach more slowly. The fast sorption occurs in the regions between the fibrils and slow sorption in the amorphous zones inside the fibrils.[2] At both kinds of sites, the water sorption follows an exponential function with a different time constant (smaller for fast and larger for slow sites) and limiting value (i.e., equilibrium moisture content at respective sorption sites).

As such, the total moisture content as a function of time (t) at a certain RH can be expressed as

$$MC(t) = MC_0 + MC_{fast} + MC_{slow}, \quad (5)$$

where $MC_0$ is constant, $MC_{fast}$ and $MC_{slow}$ are the contributions of fast and slow sorption processes, respectively, expressed by

$$MC_{fast} = EMC_{fast}\left(1 - e^{-\frac{t}{t_{fast}}}\right) \quad (6)$$

$$MC_{slow} = EMC_{slow}\left(1 - e^{-\frac{t}{t_{slow}}}\right) \quad (7)$$

where $EMC_{fast}$ and $EMC_{slow}$ represent the equilibrium moisture content (i.e., full moisture sorption capacity) at fast and slow sorption sites, respectively. Likewise, $t_{fast}$ and $t_{slow}$ express the timescales of the fast and slow sorption processes.

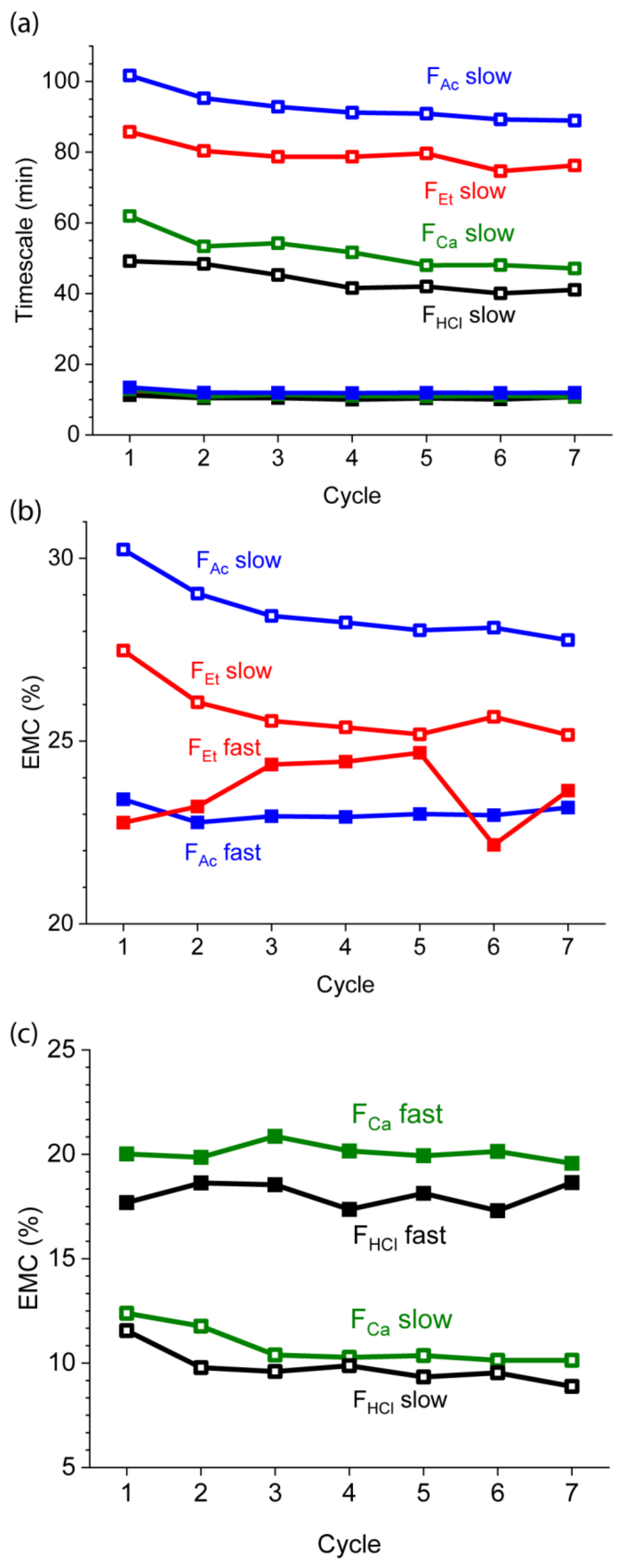


**Figure S7.** (a) time scales and (b, c) equilibrium moisture contents associated with the fast (filled symbols) and slow (empty symbols) sorption regimes for filaments coagulated in acetone (blue), ethanol (red), $CaCl_2$ (green) and HCl (black). Note that the timescales of fast sorption overlap in (a)

**Figure S7** shows the contributions of the fast and slow sorption regimes to the equilibrium moisture content in the filaments at the end of each cycle at RH 95%. Naturally, fast sorption occurs at a shorter timescale than slow sorption (**Figure S7a**). For the filaments coagulated in organic coagulants (**Figure S7b**), slow sorption (i.e., sorption at less exposed sites) is a slightly more dominant contributor to the total moisture sorption than fast one. For filaments coagulated in salt or acid solution (**Figure S7c**), this pattern is reversed and most sorption occurs at more exposed sites. In fact, when changing from organic to aqueous coagulant, EMC for the fast sorption regime decreases slightly but stays close to 20% (**Figure S7b, c**, filled symbols), while EMC for the slow one declines from >25% to <15% (**Figure S7b, c**, empty symbols).

This signifies that, on one hand, the fibril surfaces and interfibrillar spaces maintain most of their sorption behavior regardless of the coagulant. On the other hand, the sorption at the amorphous regions inside the fibrils is significantly decreased by the protonation of the carboxylic groups in acidic environment or their electrostatic screening by $Ca^{2+}$ ions. Apparently, the ion exchange acts on the carboxylate groups both on the fibril surfaces and inside the amorphous regions, influencing the water sorption behavior especially inside the fibrils.

The closure of the less accessible sorption sites in $F_{Ca}$ and $F_{HCl}$ continues upon the progress of the humidity cycling, while the sorption at the more accessible (i.e., fast) sites stays fairly at the same level (**Figure S7c**). Thus, the extensive hornification in these filaments (**Figure 7b, main article**) is mainly caused by the decline in the amount of slow sorption sites. Similarly, less exposed sorption sites are lost upon humidity cycling in the organic solvent-coagulated filaments ($F_{Ac}$ and $F_{Et}$) (**Figure S7b**). Even though they are more resistant to hornification (**Figure 7b, main article**) because of higher surface charges, their hornification is still caused mainly by the same mechanism as the filaments from the other coagulants.

As the slow sites are closing in all the samples, the remaining ones also fill up faster, which is seen as a declining timescale of the slow sorption processes (**Figure S7a**). In contrast, the fast sorption processes maintain a similar timescale during each cycle (**Figure S7a**). This finding agrees with the contribution of the fast sorption to total moisture sorption (corresponding to the amount of exposed sorption sites) staying fairly level apart from fluctuations (**Figure S7b, c**).

It should be noted, though, that the division between fast and slow sorption remains an approximation. For example, earlier authors, who examined TOCNF filaments coagulated in acetone, reported EMCs of ~25% and ~15% for fast and slow sorption regimes, respectively.[3] This follows a different trend compared to the EMCs measured in the present study: ~23% for fast and 28-30% for slow sorption. This highlights that the error margin can be large.

**Reference**


1. SAUERBREY & G. The use of quarts oscillators for weighing thin layers and for microweighing. *Z. Phys.* **155**, 206–222 (1959).
2. Kohler, R., Dück, R., Ausperger, B. & Alex, R. A numeric model for the kinetics of water vapor sorption on cellulosic reinforcement fibers. *Composite Interfaces* **10**, 255–276 (2003).
3. Lundahl, M. J. *et al.* Strength and Water Interactions of Cellulose I Filaments Wet-Spun from Cellulose Nanofibril Hydrogels. *Scientific Reports* **6**, 30695 (2016).
